# Age-specific changes in electrocardiographic parameters in bipolar limb leads of conscious female native cats of Odisha

**DOI:** 10.14202/vetworld.2016.147-150

**Published:** 2016-02-12

**Authors:** Subhashree Sarangi, A. P. K. Mahapatra, S. Mohapatra, A. K. Kundu

**Affiliations:** Department of Veterinary Physiology, College of Veterinary Science & Animal Husbandry, Odisha University of Agriculture & Technology, Bhubaneswar - 751 003, Odisha, India

**Keywords:** age-specific, conscious cat, electrocardiogram, mean electrical axis, native

## Abstract

**Aim:**

To study and compare the age-specific changes in electrocardiographic (ECG) parameters in bipolar limb leads of conscious female native cats of Odisha.

**Materials and Methods:**

20 healthy female native cats aged between 4 and 36 months of age were selected for the study. These queens were divided into two groups of 10 animals each. Group 1 constituted the queens aged 4-10 months (before puberty) with a mean weight of 2.28 kg (±0.4 standard error [SE]), and those aged 11-36 months (after puberty) with a mean weight of 3.72 kg (±0.6 SE) were in Group 2. The ECG was recorded with a 12-lead standard ECG recorder, Cardiart 108 T-MK VII-BPL (India) in the Leads I, II, and III.

**Results:**

The mean heart rates were 160.73 (±7.83) beats/min and 139.09 (±2.72) beats/min in the cats aged 4-10 months and 11-36 months, respectively. Significant differences existed among Q- and R-wave amplitudes and duration of QT-interval in Lead I. In Lead II, duration of QT and RR intervals, duration of ST-segment and amplitudes of P- and R-waves varied significantly. Significant differences were also observed in the P- and R-wave amplitudes in Lead III. The mean electrical axis was 63.11 (±1.98°) and 50.03 (±4.86°) in the cats aged 4-10 months and 11-36 months, respectively.

**Conclusion:**

Since the ECG studies on conscious cats are scanty, and the number of native cats being presented in veterinary hospitals and clinics has increased drastically, there is a need to establish the reference values for ECG parameters in conscious native cats so that the cardiovascular abnormalities can be predicted.

## Introduction

The domestic cat has a long history as a research subject; stray cats were used as research subjects by Claude Bernard and others during the early years of experimental medicine, their small size and availability making them popular candidates for both medical and surgical explorations. These have been used extensively for neurological research, brain and vision studies as well as for toxicology and endocrinology experiments. Moreover, these animals serve as excellent companion animals and are susceptible to many diseases, of both infectious and non-infectious origin.

Prediction and evaluation of cardiac diseases, particularly in small animals, is vital and this is possible with the use of an electrocardiograph. Electrocardiogram (ECG) is the recording of the potentials generated by the electrical current due to the passage of cardiac impulses through the heart. The ECG studies on cats are scanty, and those made earlier have focused mainly on anesthetic protocols [1] and a recent one on breed [2]. Some studies have also been reported on breed and age in dogs [3,4], goats [5], and sheep [6], the relation between ECG parameters and ion channels in dogs and rabbits [7,8], asphyxic episodes in rats [9], and anesthetic protocols in dogs [10].

Previous studies have been centered on the Lead II parameters only, and the purpose of this study was to expand the knowledge on the Leads I and III also. Few studies have revealed that the cardiac physiology of cats during the early life differs from that of adults [4,11], which might be due to differences in autonomic innervations of the heart and vasculature, resulting in marked differences in baroreflex control of circulation [12].

ECG parameters are not affected by gender difference [13], and puberty generally occurs between 5 and 9 months in queens [14]. Therefore, the aim of our experiment was to study and compare the age-specific changes in ECG parameters in Leads I, II, and III of conscious female native cats of the region.

## Materials and Methods

### Ethical approval

The experiments were conducted as per the guidelines laid down by the Institutional Ethical Committee and in accordance with the country law. The ECG was recorded as per standard procedure without causing any discomfort to animals.

### Animal groups

20 healthy female native cats aged between 4 and 36 months of age were selected for the study. The animals were judged healthy on the basis of history, physical examination, and ECG. These queens were divided into two groups of 10 animals each. Group 1 constituted the queens aged 4-10 months (before puberty) with a mean weight of 2.28 kg (±0.4 SE), and those aged 11-36 months (after puberty) with a mean weight of 3.72 kg (±0.6 SE) were in Group 2.

### Study method

**Figure-1:**
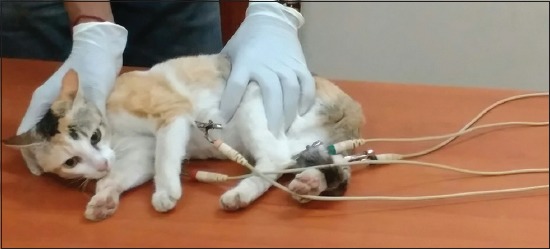
Position while recording of the electrocardiogram in a conscious female native cat of Odisha.

The ECG was recorded with a 12-lead standard ECG recorder, Cardiart 108 T-MK VII-BPL (India) in the Leads I, II and III. The ECG was set for a paper speed of 25 mm/s and sensitivity of 1 (1 cm=1 mV) with the filter (50 Hz) turned “on.” The ECG was recorded with the cat in standard body position; restrained in right lateral recumbency (Figure-1). The right forelimb and left forelimb electrodes were placed proximal to the olecranon on the caudal aspect of the respective forelimbs while the right hind limb and left hind limb electrodes were placed over the patellar ligament on the anterior aspect of the respective hind limbs using alligator clips after applying a little cardiac gel [15].

### Statistical analysis

The data obtained for each parameter in both the groups were tabulated and analyzed statistically by t-test to find out the mean and SE. A significance level of 5% (P<0.05) was adopted to compare the mean values of both the groups. The results are presented in table.

## Results

**Figure-2:**
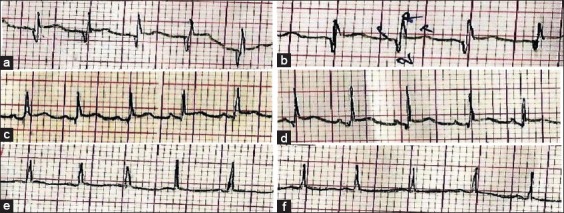
Electrocardiograms recorded in bipolar limb leads of conscious female native cats of Odisha, (a) 4-10 mon, L-I, (b) 11-36 mon, L-I, (c) 4-10 mon, L-II, (d) 11-36 mon, L-II, (e) 4-10 mon, L-III and (f) 11-36 mon, L-III.

The ECG was recorded with the typical P-QRS-T-waves each time (Figure-2). P-wave represents the depolarization of atrial muscle. Depolarization of the ventricles is represented by the QRS complex, which is followed by repolarization of the ventricles, represented by the T-wave. ECG intervals are clinically very useful. The P-R interval, measured from the beginning of the P-wave to the beginning of the QRS complex, represents the time required for the wave of excitation to travel from the sinoatrial node to the ramifications of the His–Purkinje system. The duration of the QRS complex represents the spread of impulses throughout ventricular muscle and is a measure of the intraventricular conduction time. The Q-T interval, measured from the beginning of the Q to the end of the T-wave, reflects the approximate duration of ventricular systole and the ventricular refractory period. The P-R, S-T, and T-P segments are periods of electrical inactivity during which no deflections are observed with respect to the baseline; however, P-R and S-T segments are clinically important since they occur between the waves of a single cardiac cycle. The dominant direction of electrical forces is given by the mean electrical axis (MEA).

The reference values (mean ± SE) of ECG parameters varying significantly (P<0.05) in Leads I, II, and III are shown in Table-1. The mean heart rates were 160.73 (±7.83 SE) beats/min and 139.09 (±2.72 SE) beats/min in the cats aged 4-10 months and 11-36 months respectively. Significant differences existed among Q- and R-wave amplitudes and duration of QT-interval in Lead I. In Lead II, duration of QT and RR intervals, duration of ST-segment and amplitudes of P- and R-waves varied significantly. Significant differences were also observed in the P- and R-wave amplitudes in Lead III. The MEA was 63.11 (±1.98 SE)° and 50.03 (±4.86 SE)° in the cats aged 4-10 months and 11-36 months, respectively.

**Table-1:** Reference values (mean±SE) of electrocardiographic parameters varying significantly (P<0.05) in bipolar limb leads of conscious female native cats of Odisha (rest are not significant).

Parameters	Group 1	Group 2
Lead I		
Duration (s)		
QT interval	0.11±0.014	0.16±0.014
Amplitude (mV)		
Q-wave	0.3±0.031	0.18±0.037
R-wave	0.54±0.024	0.66±0.05
Lead II		
Duration (s)		
QT interval	0.15±0.014	0.2±0.012
RR interval	0.39±0.008	0.44±0.0001
ST segment	0.03±0.014	0.08±0.012
Amplitude (mV)		
P-wave	0.18±0.02	0.12±0.02
R-wave	0.62±0.048	0.44±0.024
Lead III		
Amplitude (mV)		
P-wave	0.12±0.013	0.17±0.015
R-wave	0.46±0.081	0.22±0.037
Heart rate (beats/min)	160.73±7.83	139.09±2.72
MEA (°)	63.11±1.98	50.03±4.86

MEA=Mean electrical axis, SE=Standard error

## Discussion

Previous studies have reported that heart rate values range from 160 to 240 beats/min in normal healthy cats [16]. The parasympathetic system is predominant in adults resulting in a lower heart rate than the younger ones who possess a dominant sympathetic system [15]. Again, puberty generally occurs between 5 and 9 months for queens [14]. Cardiovascular activities are reported to be affected by different levels of sex hormones such as estradiol and dihydrotestosterone [7]. Therefore, it may be concluded that the heart rate is higher in cats aged 11-36 months due to increased estrogen levels, and this is in agreement with an experiment on dogs where heart rate decreased in the absence of sex hormones [8].

QT-interval results obtained in the Leads I and II are compatible with a study on rabbits which suggests that estrogen lengthens the Q-T interval in sexually mature female dogs [8]. R-R interval was found to be greater in adult cats which may be due to changes in QT-RR relationships from young to adult stages [17]. No literature was available on the duration of S-T segment and amplitudes of P-, Q-, and R-waves. However, the MEA values were quite lower than those reported in kitten and adult Angora cats [3], which might be due to differences in breed characteristics, i.e., shape and size of the heart.

## Conclusion

Since the ECG studies on conscious cats are scanty, and the number of native cats being presented in veterinary hospitals and clinics has increased drastically, there is a need to establish the reference values for ECG parameters in conscious native cats. ECG is an important diagnostic tool to detect and evaluate many kinds of cardiovascular abnormalities or arrhythmias such as atrial fibrillation, ventricular tachycardia, conduction block, and premature atrial and ventricular complexes, and diagnosis would be possible only if the normal values are established for the breed.

## Authors’ Contributions

This study was planned by SS and AKK. SS carried out the research under the guidance of APKM and SM. All the authors participated in draft and revision of the manuscript. All the authors read and approved the final manuscript.
